# *In Silico* Screening Based on Predictive Algorithms as a Design Tool for Exon Skipping Oligonucleotides in Duchenne Muscular Dystrophy

**DOI:** 10.1371/journal.pone.0120058

**Published:** 2015-03-27

**Authors:** Yusuke Echigoya, Vincent Mouly, Luis Garcia, Toshifumi Yokota, William Duddy

**Affiliations:** 1 University of Alberta, Faculty of Medicine and Dentistry, Department of Medical Genetics, Edmonton, Alberta, Canada; 2 UPMC—Sorbonne Universités—Univ. Paris 6, UPMC/INSERM UMRS974, CNRS FRE 3617, Center of Research in Myology, Paris, 75651 cedex 13, France; 3 UFR des Sciences de la Santé, Université de Versailles Saint-Quentin-en-Yvelines, 78180 Montigny-le-Bretonneux, France; 4 Muscular Dystrophy Canada Research Chair, University of Alberta, Edmonton, Alberta, Canada; International Centre for Genetic Engineering and Biotechnology, ITALY

## Abstract

The use of antisense ‘splice-switching’ oligonucleotides to induce exon skipping represents a potential therapeutic approach to various human genetic diseases. It has achieved greatest maturity in exon skipping of the dystrophin transcript in Duchenne muscular dystrophy (DMD), for which several clinical trials are completed or ongoing, and a large body of data exists describing tested oligonucleotides and their efficacy. The rational design of an exon skipping oligonucleotide involves the choice of an antisense sequence, usually between 15 and 32 nucleotides, targeting the exon that is to be skipped. Although parameters describing the target site can be computationally estimated and several have been identified to correlate with efficacy, methods to predict efficacy are limited. Here, an *in silico* pre-screening approach is proposed, based on predictive statistical modelling. Previous DMD data were compiled together and, for each oligonucleotide, some 60 descriptors were considered. Statistical modelling approaches were applied to derive algorithms that predict exon skipping for a given target site. We confirmed (1) the binding energetics of the oligonucleotide to the RNA, and (2) the distance in bases of the target site from the splice acceptor site, as the two most predictive parameters, and we included these and several other parameters (while discounting many) into an *in silico* screening process, based on their capacity to predict high or low efficacy in either phosphorodiamidate morpholino oligomers (89% correctly predicted) and/or 2’O Methyl RNA oligonucleotides (76% correctly predicted). Predictions correlated strongly with *in vitro* testing for sixteen *de novo* PMO sequences targeting various positions on *DMD* exons 44 (R^2^ 0.89) and 53 (R^2^ 0.89), one of which represents a potential novel candidate for clinical trials. We provide these algorithms together with a computational tool that facilitates screening to predict exon skipping efficacy at each position of a target exon.

## Introduction

Control of splicing as a therapeutic approach is currently being explored in breast cancer [1], chronic inflammatory diseases [2], and various neuromuscular conditions, including Duchenne muscular dystrophy (DMD; reviewed [3]), spinal muscle atrophy, limb girdle muscular dystrophy type 2A, hypertrophic cardiomyopathy, Fukuyama muscular dystrophy, and a severe form of core myopathy [4–9]. Diseases of RNA mis-splicing comprise up to 15% of all inherited diseases, and 50–60% of all disease-causing mutations are now known to affect splicing [10]. Splice-switching molecules are potentially applicable to these and to diseases in which recovery of a mutated gene’s open reading frame by ‘exon skipping’ could result in truncated but still functional protein. Such is the case for DMD, for which the exon skipping approach has emerged as a promising therapy showing limited success with systemic administration of two different chemistries (phosphorodiamidate morpholino oligomer, PMO; and 2’O-Methyl RNA) in clinical trials [11–13].

DMD is a severe X-linked neuromuscular disorder with incidence estimated at 1 in 3,500 male births [14]. Muscle weakness evident from around 3 to 5 years of age progresses to loss of ambulation in early to mid-teens [15]. Advances in respiratory intervention are pushing life expectancy to beyond 40 years, with a coincident shift in the cause of death away from respiratory failure and towards dilated cardiomyopathy [16]. DMD is caused by loss of the dystrophin protein, the muscle isoform being encoded by all 79 exons of the longest known human gene (*DMD*; 2.2 mega base pairs). Mutations, approximately one-third of which are *de novo*, occur in great variety, being scattered throughout the full length of this gene. Study of murine models suggests that lack of dystrophin predisposes myofibres (long, multinucleate, contractile cells comprising the bulk of skeletal muscle) to necrosis, prompting recurrent rounds of myofibre degeneration and regeneration, these exhausting the self-renewal capacity of the native precursor population as it struggles to compensate for the cellular pathology [17–19]. As patients age, their lean muscle mass diminishes, and is partially replaced by fibrotic tissue. Despite a number of approaches, including cell and gene therapies, upregulation of the dystrophin homologue utrophin, gene editing [20,21], and stop codon read-through, therapies to slow disease progression have remained elusive (recent reviews: [22–25]).

Exon skipping therapy for DMD aims to shift the clinical prognosis towards its milder counterpart Becker muscular dystrophy (BMD), by interfering with splicing events using agents such as antisense oligonucleotides (referred to here as oligos). The diverse severity of clinical outcomes that result from the wide variety of different *DMD* mutation patterns is explained in large part by the “reading frame rule” [26,27], pertaining to the alignment or mis-alignment of exon boundaries with codon triplets. A mutation, such as the deletion of a given exon or exons, may change the downstream open reading frame, leading to a premature stop codon and nonsense-mediated decay of the RNA transcript: such mutations usually result in DMD. Conversely, the mutation may leave the open reading frame unchanged, allowing the translation of a truncated protein that, if it retains certain functionalities, gives rise to the milder BMD. Exon skipping seeks to disrupt the definition of an exon adjacent to the mutation such that it is not spliced into the mRNA transcript and the correct open reading frame is restored.

Efficient exon skipping depends on the interaction of a given oligo with the complex system represented by the splicing apparatus and regulatory factors, the nascent pre-mRNA, and the interactions of these components with each other and with the rest of the cellular environment. Efficacy varies markedly between different target sequences on the exon that is to be skipped. Studies have generally involved the *in vitro* testing of many target sequences for a given exon, with no guarantee that the eventual target sequence selected represents an optimal choice for *in vivo* efficacy.

Design guidelines have been put forwards [28–32] that identify several important parameters based on their correlation with observed efficacy. Three large-scale studies tested the efficacy of many oligonucleotides and retrospectively identified correlating parameters [28,29][31]. Aartsma-Rus *et al*. compared the efficacy of targeting exonic sequences against that observed for targeting splice sites at the exon/intron boundaries, finding that the former were more efficient, in large part due to the greater GC content of the exon (and therefore superior thermodynamic binding of oligos targeting the exon) [31]. Aartsma-Rus *et al*. furthermore [28] used linear discriminant analysis to identify four parameters capable to classify a high proportion of 2’O-Methyl oligos into effective and non-effective categories (defined by those authors as >5% or <5% of transcript skipped). The strongest of these was the difference in free energy (dG) between the bound and unbound states of the oligo and the entire sequence of the exon. The remaining three parameters related to the binding of splice regulatory proteins to the target sequence within the exonic pre-mRNA: the number of hexameric exon splicing enhancer sites predicted by RESCUE-ESE [33], and the strength of sequence motifs for the binding of Tra2β and SC35 splicing factor proteins as predicted by Human Splicing Finder [34]. Popplewell *et al*. [29], carrying out a similar analysis but using PMO oligos, likewise identified (1) dG of the oligo to its target exon, but otherwise determined (2) oligo length, (3) the proximity of the target sequence to the exon acceptor site, (4) their experimental assay of target site accessibility, and (5) the predicted strength of ASF/SF2 splice factor binding, to be the other distinguishing parameters. A purely *in silico* analysis of RNA accessibility identified parameters based on the computation of co-transcriptional folding dynamics [35]. Their algorithm computed the free folding of a moving window of 1500 bases of the pre-mRNA, beginning with the exon at the 3’ end of the window then shifting one base per iteration until the exon was at its 5’ end. The number of times that bases of the target sequence were unbound throughout the iterations was computed, and was found to correlate with previously described skipping efficacy. A prospective study was later carried out using, as its oligo design criteria, a parameter (‘L3’) that was based on these RNA accessibility computations, together with two other parameters: the presence of exon splice enhancer motifs and the oligo length [30].

These previous studies involving retrospective identification of design parameters were limited to single-study datasets and did not set specific criteria or thresholds [28,29], whereas the prospective study [30] considered only several design criteria from among those parameters that had previously been identified to correlate with efficacy. Importantly, no reports have moved beyond the identification of design criteria, to provide quantitative predictions of skipping efficacy. Furthermore, the retrospective analyses may, in the absence of checking against other datasets, have yielded potentially spurious study-specific biases in correlation, especially considering the large numbers of parameters involved. In addition, there are two contexts in which consideration of RNA folding and binding energetics have been based on assumptions that do not necessarily reflect as closely as possible the dynamics of the system: (1) because binding of the oligo occurs during the definition of the exon, it may make more sense to consider binding to a target region within which the target sequence is at the center (including where necessary some intronic sequence) rather than binding to the entire exon, which could be particularly inaccurate for oligo binding near the ends of the exon; (2) the pre-mRNA may have strong limitations on its freedom of movement over a window as long as 1500 bases, so computations of RNA accessibility over shorter lengths may give greater accuracy. Even in a free-folding system, the limitations of computational accuracy should be considered: for example, it was recently shown that a length of 150 bases provided a balance between maximizing the number of accurately predicted base pairs, while minimizing the effects of incorrect long-range predictions [36]. Besides energetics, various other parameters have not yet been considered, including (1) the estimation of splice factor binding propensity to a given sequence based on sequence neighborhood inference [37], (2) categorization of the target exon according to splice-related characteristics (based on the machine learning analysis by [38]) and (3) differential GC content of the flanking intronic sequence with its exon, which was recently shown to be important for exon definition in a subset of human genes [39], including (shown here) the *DMD* gene.

Here, we present a novel approach for screening across an exon to predict regions of high exon skipping efficacy. We collate previously published data on the efficacy of exon skipping oligos and their reported descriptive parameters. For each previously published oligo we then use computational tools to calculate additional parameters, usually with several variations of each (such as varying the length of flanking sequence in computations of binding energy). We identify parameters that have robust predictive power and, based on these, derive predictive algorithms and apply them to screen across all target positions within selected exons of the dystrophin pre-mRNA. Predictions are then tested by *in vitro* assays of exon skipping for oligos at eight positions each of dystrophin exons 44 and 53, and a strong correlation is found with the predicted profile across the exon. Somewhat surprisingly, although several parameters were additive to predictive power in the PMO and 2’O-Methyl datasets, none were shared by both datasets except for (1) the binding energy of the oligonucleotide to the RNA, which was found to have the strongest influence on efficacy, and (2) the distance (in bases) of the target site from the splice acceptor. An *in silico* screening tool facilitating the application of these algorithms across any exon sequence of interest is provided as a Perl script with instructions for use.

## Materials and Methods

### Parameter calculations and predictive modelling

Binding energy for the oligo to the target site was computed using the RNAeval algorithm with its default settings [40]. Energy of binding to the target with flanking regions of various extents was computed twice, using either RNAstructure v5.3 [41] or RNAcofold [40]. Results from RNAstructure and RNAcofold were found to correlate closely and those from RNAstructure were used for modelling. Scores for RNA accessibility were generated using the RNAplfold algorithm [40]. From its output, custom Perl programs retrieved accessibility scores for each published target sequence, its last 15 bases (i.e. 15 bases counting inwards from the 3’ end), its last 8 bases, and each of its bases singly, from which were derived our various accessibility descriptors. For neighborhood inference scores, a Perl program was written to screen across all target exons and, for each hexamer sequence, retrieve the NI score published by [37]. The cumulative NI score was defined as the sum of the hexamers scores for each target site. We also divided this cumulated score by target length to give a normalized cumulative NI score. GC content of the exon, the upstream flanking region, and our various secondary parameters based on those, were calculated using Perl and BioPerl. Data exploration and modelling were done using JMP software (SAS Institute Inc., NC, USA). Further details relating to parameter calculations and predictive modelling can be found in supplemental materials and methods.

### Screening of predictive algorithm across target exons

Human *DMD* exon coordinates were taken from the Consensus CDS project reference CCDS14233.1 and sequences were downloaded from NCBI nucleotide reference NC_000023. A fasta file was generated for target exons and 200 base intronic flanks (S1 Sequences). We include a Perl script (S1 Script) which takes the fasta file as input and generates two output files: (1) a list of oligo and target sequences (with flanking regions) for every position of the target exon(s), numbered according to the distance of the target sequence from the splice acceptor site, and (2) energy calculations for each of these oligo to target pairs. The script uses BioPerl’s sequence input/output object to manipulate the fasta file, and the text interface version of RNAstructure software [41] to generate energy calculations. The script has a path to RNAstructure thermodynamic parameter tables that must be set correctly and RNAstructure must be correctly installed on the system. The length of the oligo sequence can be specified in the script, along with several other parameters. The predictive formulae (PMO or 2’O-Methyl) presented here can then be applied to each target position, using Microsoft Excel to incorporate the dG and distance from acceptor values output by RNAstructure and Perl into the formula.

### Prospective testing

Human-derived cell lines were obtained in collaboration with the team of F Muntoni, MRC CNMD Biobank (NHS Research Ethics Committee reference 06/Q0406/33; and HTA license number 12198), in the context of Myobank, affiliated to Eurobiobank (European certification). Antisense PMO oligonucleotides of length 30bases targeting *DMD* exon 44 or 53 were synthesized (Gene Tools). Human-derived myoblasts from two DMD patients (immortalized clonal lines 7796 and KM571 [42]) harboring, respectively, deletion of exons 42–43 and of exon 52 of the *DMD* gene, and therefore having an open reading frame correctable by skipping (respectively) of exon 44 or 53, or from a healthy subject (immortalized clonal line 8220) were cultured in proliferation conditions with growth medium (GM) (DMEM/F12 with skeletal muscle supplement mix (Promocell), 20% FBS and 0.5% antibiotics (penicillin/streptomycin). Cells were seeded at 0.6 x 10^5^ per well in a 12-well plate coated with collagen type 1. Two days after seeding, at approximately 90% confluence, GM was changed to differentiation medium (DM) (DMEM/F12 with 2% horse serum, 1x ITS solution (Sigma), and 0.5% antibiotics (PC/ST). After three days in DM, PMOs at 10 μM were added in individual wells, with 6 μM of end-porter transfection reagent. Two days following PMO transfection, the DM containing PMO was replaced with regular DM. Cells were harvested at day 8 of differentiation (5 days after PMO transfection). Skipping percentage was calculated as $\frac{\text{skipped transcript}}{\text{skipped transcript}+\text{native transcript}}$ using ImageJ software (NIH). In exon 44 skipping, an unknown top band above the native band, and an intermediate band, were excluded from the RNA quantification calculation. For western blots, anti-alpha-tubulin antibody was used as a loading control (ab7291, 1:8000, Abcam). Expression levels of dystrophin protein in the DMD cells transfected with PMOs were calculated with a calibration curve from 1 to 10 percent protein (0.12–1.2 μg) of immortalized healthy skeletal muscle cells at 9 days after differentiation was used as a positive control. Three independent experiments were performed for each oligonucleotide tested. Uncropped images of all gels and blots are provided (S6 Fig.). Further details of RT-PCR and western blots are given in supplemental materials and methods.

## Results

### Database creation: curation of existing data on previously tested sequences

Survey of the literature identified five large-scale studies in each of which tens of oligonucleotide sequences had been tested for exon skipping [28–30,43–45]. These studies differed in the oligonucleotide chemistries and cell types used, and in their reporting of skipping efficacy information and of associated experimental details. Some reported various computed parameters including physical descriptions of the oligo (e.g. length, percentage GC content), binding energies (oligo to its target sequence, or oligo dimerization), the situation of the target sequence (distance from splice acceptor and/or donor site), and local target site characteristics (predicted openness or accessibility of target site conformation; predicted binding of splicing factors). Skipping efficacy was always based on the ratio of skipped to native transcript observed following nested reverse transcription polymerase chain reaction (nested PCR) analyses, and was reported either as a percentage $\frac{\text{skipped transcript}}{\text{skipped transcript}+\text{native transcript}}$ or as a percentage classed into one of 2 to 4 grades (e.g. grade 1: <10%; grade 2: 10–30%; and grade 3: >30%). In general, the authors of these studies defined a ‘good’ level of exon skipping to be greater than 25–30%. A summary of the studies used is shown (table 1). These were combined to create two datasets (one for PMO oligos, and one for 2’O-Methyl oligos) detailing a total of 358 oligonucleotides, with care taken to correctly interpret the different numbering systems used by each study (S1 and S2 Tables). Where absent in the original publication, basic descriptors such as the length of the oligo, or the distance of the target site from the exon acceptor site, were calculated. As a descriptor of target site situation that is independent of oligo length, we used the ‘Average Cumulative position’ (ACP; essentially the distance in bases from the splice acceptor site to the centre of the target site) defined by [30], but retained descriptors for the distance of the oligo’s extreme bases to their respective exon acceptor and donor sites.

**Table 1:** *Skipping efficacy was always based on the ratio of skipped to native transcript observed following nested PCR analyses, and was reported either as an exact percentage ((skipped transcript)/(skipped transcript + native transcript)) or as this percentage classed into one of 2 to 4 grades (e.g. grade 1: <10%; grade 2: 10–30%; and grade 3: >30%).

Study	Chemistry	Method of reporting efficacy*	# oligos
Aartsma-Rus 2005 & 2009	2’ O-Methyl RNA	>25%, 1–25%, and undetected or >5% and <5%^†^	156
Dwi Pramono 2012	2’ O-Methyl RNA	Exact percentage	23
Harding 2007	2’ O-Methyl RNA	>30%, 10–30%, 1–10%, and 0%	34
Popplewell 2009	PMO	Exact percentage	66
Wilton 2007	2’ O-Methyl RNA	>30%, 10–30%, and <10%	79
		**Total**	**359**

^†^Levels of efficacy were reported as >5% or <5% for 44 oligos of the Aartsma-Rus data.

### Additional descriptors

We wished to take a systematic approach, including into our analysis many factors that were calculable or predictable by way of calculation and that might contribute to skipping efficacy. We supplemented previously reported descriptors with additional parameters (summarized in table 2). All 358 oligos and their descriptor values (published and additional) are given in S1 and S2 Tables. The additional parameters fell into four categories: (1) binding energetics of the oligo to its target region; (2) predicted accessibility of the target site; (3) predicted splicing motifs at target site; and (4) characteristics of the target exon and the upstream intron. Although some of these parameters (e.g. the exon GC content) do not change between different target sites within the same exon, they could still influence the interplay of other factors (e.g. the importance of binding energy could be relatively weak in exons of high GC content).

**Table 2:** Summary of additional descriptive parameters.

Predicted energetics	Predicted accessibility	Predicted splicing motifs	Target exon and the upstream intron
**Binding energy (dG) of oligo to target with**: (1) 50 base flanks; (2) 100 base flanks; (3) 200 base flanks; (4) flank from exon start to 10 bases downstream of target end	**Accessibility scores for**: (1) Target region; (2) Target region (normalized to oligo length); (3) 15 bases of target 3’ end; (4) 8 bases of target 3’ end; (5) Most accessible 8 bases	**Neighborhood inference scores**: (1) Cumulative; (2) Per base	**Exon ‘Malueka’ splice category**
	**Co-transcriptional folding**: (1) L1 score; (2) L3 score		**Exon/intron GC content**: (1) Exon GC%; (2) Intron GC%; (3) ΔGC; (4) ΔGC (ignoring target); (5) Change in ΔGC after oligo binding

**Fig 1:**
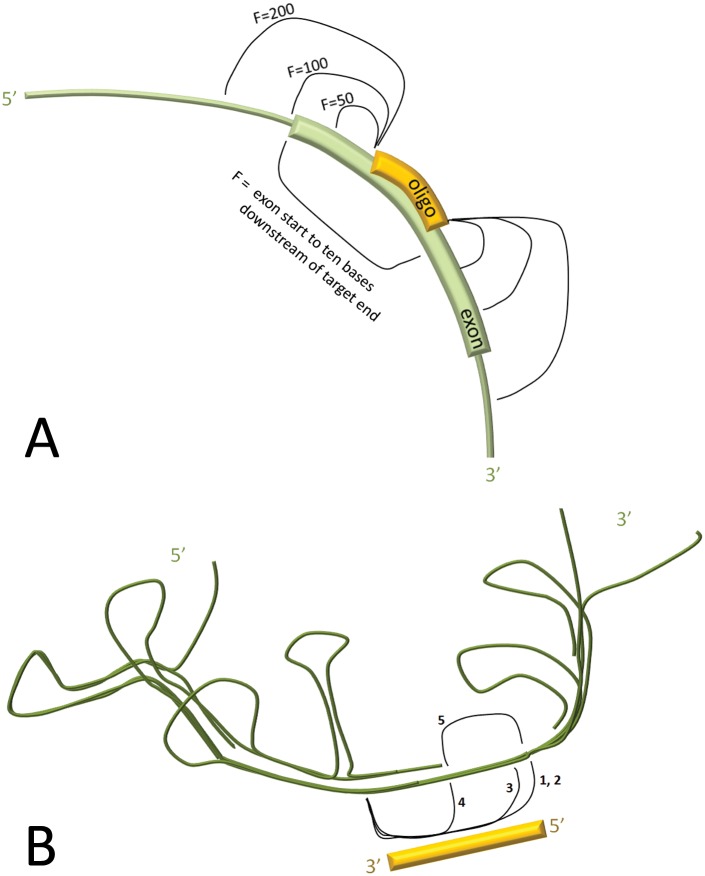
Binding energetics and RNA folding accessibility. (A) The energy (dG) of binding was calculated for the oligo to the target region and flanking regions of various extents: flanks of 50 (dG50), 100 (dG100), or 200 (dG200) bases around the target site, or flanks extending from the 5’ end of the exon to 10 bases downstream of the target 3’ end (dG_et+10_). (B) Accessibility (the likelihood of bases being unpaired) of the target site within the predicted structure of the folded RNA: (1) target site; (2) target site normalized to oligo length; (3) the 15 bases of the target’s 3’ end; (4) the 8 bases of the target’s 3’ end; (5) the most accessible 8 bases of the target site.

(1) Binding energetics of the oligo to its target region: Because binding energy is dependent not only on direct contacts between the oligo and its target sequence, but also on binding of the target region (the target and its surrounding sequence) with portions of itself, we calculated the dG of binding for the oligo to the target plus flanking regions of various lengths (50, 100, and 200 bases). Also, imagining that the upstream portion of the exon may have time to adopt some local folding prior to its exposure to the oligo, we calculated the dG of binding for the oligo to the target plus flanking regions extending upstream until the exon start and downstream by 10 bases from the target site end. These calculations are represented in Fig. 1A.

(2) Predicted accessibility of the target site: The accessibility of each base in a RNA sequence can be predicted by thermodynamics calculations and may influence the binding efficacy at the target site. It has been shown in siRNA studies that target site accessibility correlates with efficacy [46], and particularly the accessibility of bases towards the 3’ end of the target site [47]. Specifically, for a test dataset of oligos targeted across various regions of mRNAs, the accessibility of the 8 base pairs at the 3’ end of the target site gave the strongest association with efficacy. Since target site accessibility will also influence exon skipping efficacy, we calculated accessibility for each base of each target exon. From this we derived several different accessibility scores by summing across bases at the following sites: (1) the target; (2) the target (score normalized to oligo length); (3) the 3’ end of the target (last 15 bases); (4) the 3’ end of the target (last 8 bases); (5) the site of maximum accessibility within the target (the eight contiguous bases of the target having the greatest summed accessibility scores). These are represented in Fig. 1B.

In addition, an attempt has been made to use thermodynamics calculations to model co-transcriptional pre-mRNA folding [35]. The authors represented folding by defining a window of 1500 nucleotides and moving this window in single base increments over a region anchored by the exon skipping target site. For each increment of the window they computed the RNA secondary structure, allowing them to measure the proportion of structures for which a given base is bound or unbound, and thereby to derive an ‘engagement’ score for that base. We included into our database, scores for (L1) the average proportion of predicted structures for which each target base is unbound, and (L3) the average engagement of the target bases during virtual transcription, that had been calculated by [35] for two of the exon skipping studies [43] and [45].

(3) Predicted splicing motifs at target site: Work by [37] enables a generalized approach to the estimation of splice factor binding propensity to a given sequence. These authors correctly predicted novel ESE and ESS sites based on Neighborhood Inference (NI) scoring, which examines the relationship of all known splice factor binding motifs to other regions in sequence space (the mathematical space that describes all possible sequences and their similarity to one another). Their publication includes a list of all possible hexamer sequences with a NI score calculated for each, which we used to derive (1) a cumulative NI score (sum of all hexamer scores), and (2) the cumulative NI score normalized to target sequence length, for each exon skipping target site.

(4) Characteristics of the target exon and the upstream intron: exon splice factor properties and exon/intron GC contents: Exons differ in various properties that may influence splicing and thus the efficacy of exon skipping. Machine learning algorithms were recently applied [38] to categorize the exons of the *DMD* gene on the basis of splicing-related parameters, including splice site strengths, splice site GC contents, and enrichment for different types of splicing motif. We included this exon categorization as a descriptor for each oligo.

It was recently observed that the exon/intron architecture of human and other homeothermic organisms can be placed into two categories: (1) the exon and its flanking introns have high GC content and the differential GC content between the exon and its flanks (ΔGC) is low, introns are short, and (2) overall low GC content but a high ΔGC where the exon has relatively high GC content compared with its flanking introns, introns are long [39]. The authors presented various bioinformatics and experimental validations supporting the hypothesis that the high ΔGC in type 2 architecture was important for the definition of the exon during splicing. We noted that the exon/intron architectures of the *DMD* gene belong to this second category, having long introns, an average intronic GC content of 32%, and average exonic GC content of 44% (S2 table). Since the GC content differential might affect exon definition and thus influence skipping efficacy, and because the nuances of this differential might be affected by oligo binding, we calculated several parameters including: (1) exon GC content; (2) GC content of the upstream intron (150 bp); (3) ΔGC; (4) ΔGC when the target site is ignored (supposing some blocking effect of the bound oligo); (5) change in ΔGC due to oligo blocking. We calculated ΔGC based on the upstream intron because this was more pronounced in the study by [39].

### Predictive statistical modelling

We carried out preliminary data exploration to select only a subset of each parameter category to apply forward into predictive modelling, and we also tested the cross-terms describing relationships between whole exon descriptors (e.g. the GC contents of the exon and upstream intronic sequence) and target site descriptors. We found that an energetics parameter, dG50 (the energy of oligo binding to the target plus 50 base flanks), showed strong correlation with efficacy in the two larger datasets we investigated (Aartsma-Rus and Popplewell), and for modelling we used this parameter in favour of other energetics parameters such as the binding of the oligo to its target site only or to the entire exon sequence. The dG50 was considerably more predictive than these other parameters for the Aartsma-Rus dataset (p = 3 x 10^-4^ for dG50, 0.01 for oligo::target, and 0.03 for oligo::exon) and, for the Popplewell dataset, only slightly less predictive than binding to the whole exon (p = 9 x 10^-5^ for dG50, 0.001 for oligo::target, and 1 x 10^-5^ for oligo::exon). Data for the two chemistries (2’ O-Methyl RNA and PMO) were modelled separately, with the aim to identify parameters that were robustly predictive across both chemistries. We focused our analysis on the PMO data [29] since it included many data-points (66 oligos), had good coverage of four exons (8–23 oligos tested for each of exons 44, 45, 46, and 53) and partial coverage of a fifth (2 oligos tested for exon 51), and the authors reported percentage values for efficacy that could be more powerfully leveraged in modelling than could the stratified levels of efficacy reported for 2’O-Methyl oligos.

**Fig 2:**
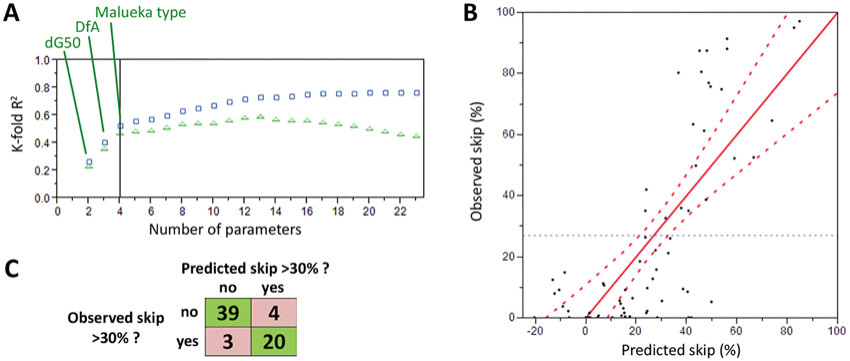
Predictive modelling for PMO oligonucleotides. (A) Descriptive parameters are added consecutively to the model based on their predictive power (except parameter 1, which is the intercept). K-fold R^2^ (green triangles), an indicator of the likelihood of the model to be predictive of new datasets, increases markedly with the addition of dG50 (binding energy of the oligo to a region encompassing the target site and 50 base flanks), DfA, the distance of the target from the upstream splice acceptor site, and Malueka type, the category of the exon based on splice-related descriptors defined by [38]. R^2^ (blue squares) is improved by the addition of further parameters but K-fold R^2^ is only slightly increased. (B) Percentage skipping as reported (Observed skip) against the percentage skipping, as predicted by a standard least squares model for the skipping efficacy of PMO oligos, based on the parameters: dG50, DfA, Malueka type, and the factorial of dG50 x DfA. A line of best fit is marked (red line) with 0.05 significance curves (dashed red lines), and average of observed (dashed blue line). (C) Confusion matrix showing the numbers of correct and incorrect predictions of skipping efficacy greater or less than 30% by an ordinal logistic model constructed using the same parameters (green background = correctly predicted; red = incorrectly predicted).

We used a modelling approach known as stepwise regression with K-fold cross-validation to identify parameters that were predictive of skipping efficacy. Parameters and their factorials were then used to construct a predictive formula that could be applied across target sites. This approach divides the original data into K equal-sized subsets, randomly partitioning each entry into a subset. In turn, each of the K sets is used to validate the model fit on the rest of the data, fitting a total of K models. The K-fold R^2^ is calculated as the average for all of the models. The R^2^ value for the correlation of the model with the data improves as parameters are added to the model, whereas the K-fold R^2^ improves only to the point where additional parameters are unlikely to have predictive value. As shown (Fig. 2A), K-fold R^2^ identified three parameters that had strong predictive power for the Popplewell dataset: (1) the dG of binding for the oligo to the target plus 50 base flanking regions; (2) the distance of the target site from the splice acceptor; and (3) the Malueka category for splice-factor related characteristics of the exon. It can be seen in Fig. 2A that the K-fold R^2^ score could be marginally increased with the addition of more parameters but we elected to be conservative in our modelling, using only these three strongest parameters, in order to diminish the likelihood of over-fitting the model to the data, which is a danger when modelling sparse datasets and can lead to a model which fails to be predictive of new data. A second round of stepwise regression on these three parameters and their factorials indicated that the inclusion of the cross-term of distance from acceptor with binding energy added to the predictive power. A standard least squares model was then constructed from which was derived a predictive formula for skipping efficacy (Fig. 2B). The model gave R^2^ of 0.57 and RMSE of 21.1 and was comprised of the following terms:

$$Predicted\;skip=-44.177+\left(-0.253\;\times DfA\right)+\;\left(-2.435\;\times \;dG50\right)+\left(\begin{array}{c} Malueka\;A\;\Rightarrow -11.4\\ Malueka\;C\;\Rightarrow \;+11.4 \end{array}\right)+\;\left(\left(DfA\;\times \;-80.197\right)\times \;\left(dG50\;+\;37.467\right)\times \;0.02\right)$$

Where: DfA = Distance of target’s 5’ end from upstream acceptor site in bases; dG50 = Energy of binding of the oligo to the target site with 50 base flanks; and Malueka A/C = Category of exon based on categorization by [38].

There were two groups of outliers, one with good predicted efficacy (30–50%) but poor observed efficacy (0–10%), the other with good predicted efficacy (40–50%) but excellent observed efficacy (70–90%); outlier characteristics were examined but no commonalities were identified.

To test the capacity of these parameters to correctly place oligos according to ‘good’ (>30%) or ‘bad’ (<30%) efficacy, an ordinal logistic model was generated, predictive of this categorization. The model correctly placed 89% of oligos, as shown by the confusion matrix (Fig. 2C).

**Fig 3:**
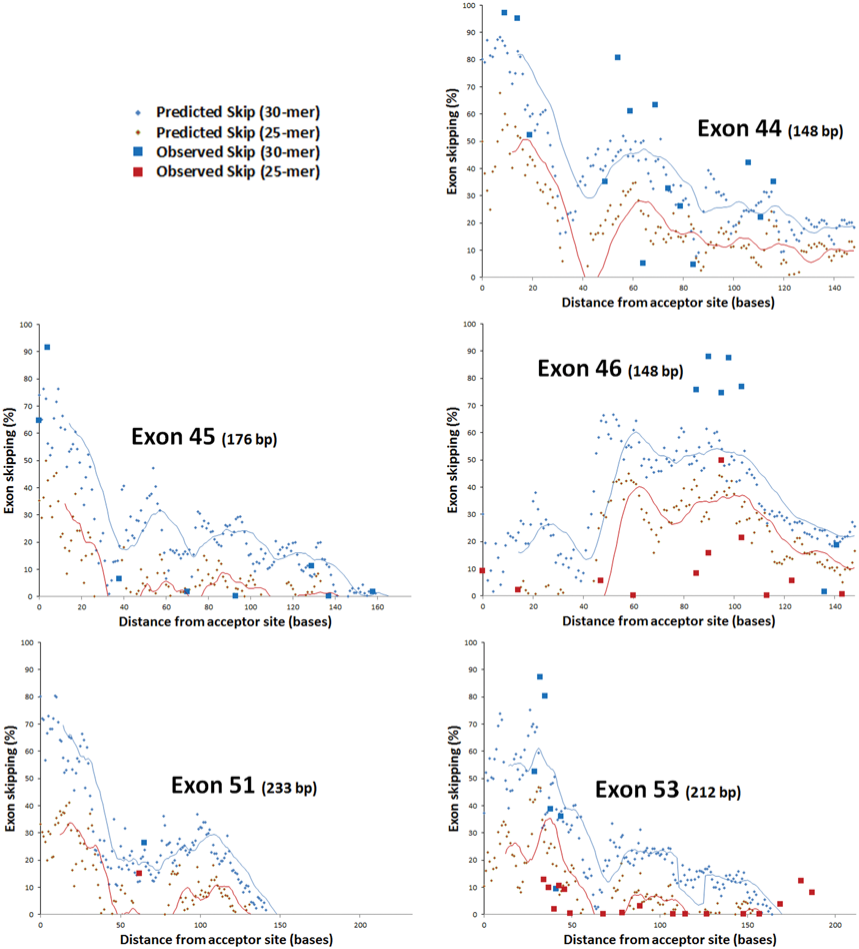
Screening across exons for predicted exon skipping and comparison with published data (from [29]). Distance from acceptor is given for the first (5’-most) base of the target site. Observed and predicted percentage skipping are shown for 30-mer and 25-mer oligonucleotides, with moving averages shown over 15 and 13 bases, respectively (in this way, the moving average indicates the value for the mid-point of each target site).

To visualize trends in predicted efficacy and to compare the predicted with the observed efficacy we created an *in silico* screening tool (see materials and methods) and applied it across the exons tested in the Popplewell study (Fig. 3), calculating predicted efficacy for 25-mers and 30-mers at every possible target position. Conformity to the model was most notable in the diminishing of efficacy with distance from the acceptor site, except when strong dG of binding counteracted this effect, as for bases 80 to 110 of exon 46. Skipping (both observed and predicted) was generally more effective in exons belonging to Malueka splice type C (exons 44 and 46), than those of type A (45, 51 and 53), especially towards the donor site end of the exon.

**Fig 4:**
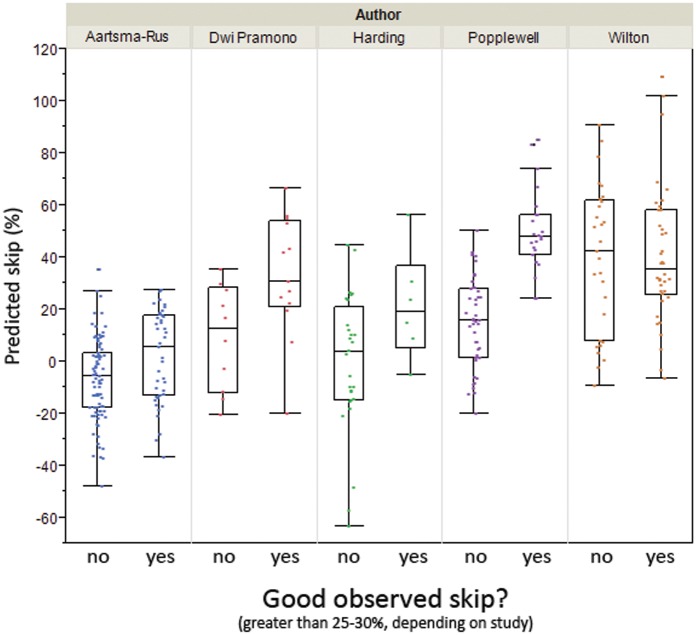
Predictive formulas derived for PMO (Popplewell) data applied across 2’O Methyl data (Aartsma-Rus, Dwi Pramono, Harding, and Wilton). Predicted skip is shown for oligos categorised as ‘good’ or not, based on the level stratifications reported for each study (Aartsma-Rus, >25%; Dwi Pramono, >27.5%; Harding, >30%; Popplewell, >30%; Wilton, >30%).

We tested whether the predictive formula derived from the PMO data could distinguish efficacious from non-efficacious oligos in the 2’O Methyl datasets (Fig. 4). Average values of predicted skip varied markedly between studies, partly following the authors’ varying choices of oligo length. Average values were lower for the 2’O Methyl studies than for PMOs because PMO oligos have lower binding affinity, requiring longer sequences. In general, efficacious oligos had higher predictions than non-efficacious oligos, except for the Wilton dataset. However, this dataset had notable peculiarities that may limit its use for predictive analyses: (1) a single most successful oligo is reported per exon; (2) efficacy for exons in the range 55–70 was dramatically diminished relative to other exons (S1 Fig.). Among our test parameters we could find none that would explain this latter observation, and we opted in subsequent analyses to omit this dataset.

To identify the factors influencing skipping efficacy in the 2’O Methyl datasets we carried out stepwise regression. As for the PMO dataset, dG of binding of the oligo to the target site, and distance from the acceptor site (or ACP, which gave very similar results) were strongly predictive parameters, and it was useful to also include their cross-term. Unlike the PMO dataset, an oligo length parameter (categorizing into > 22 or < 22 bases) was selected, whereas the exon Malueka splice type was not. These same parameters were used to generate an ordinal logistic model that correctly placed 75.6% of 2’O Methyl oligos into ‘good’ or ‘bad’ efficacy (defined as greater or less than 25%, 27.5%, or 30% skipping, depending on the stratification of skip levels reported in the study; it was necessary here to omit 28 oligos from the Aartsma-Rus data, for which reported efficacies of >5% were not further stratified) (table 3). Correct placing was better for the Harding (79.4%) and Dwi Pramono (82.6%) datasets than for the Aartsma-Rus dataset (73.4%). We applied the predictive formula for 2’O Methyl in an *in silico* screen across the five exons that have been most frequently targeted using 2’O-Methyl oligos (exons 44, 45, 46, 51, and 53; S3 Fig.). Agreement with observation was strong for exons 51 and 53, but poor for exon 45, which may be explained by the coverage of this exon (and also of exon 46) primarily by very short oligos (15–18 nucleotides) which generally had a high rate of failure (S4 Fig.). The predictive formula for screening 2’O Methyl RNA target sites is given (S2 Fig.).

A complete list is given of the sixty parameters considered, indicating which were included into the two predictive algorithms (S3 Table).

**Table 3:** ^†^Predicted skip is shown for 2’O-Methyl oligos categorised as ‘good’ or not, based on the level stratifications reported for each study (Aartsma-Rus, >25%; Dwi Pramono, >27.5%; Harding, >30%).

Study	Observed ‘good’ skip?^†^	Predicted ‘good’ skip?^†^		Proportion predicted correctly
All 2’ O Methyl		no	yes	
	no	111	14	
	yes	31	29	75.6%
Aartsma-Rus^**†**^		no	yes	
	no	77	10	
	yes	24	17	73.4%
Dwi Pramono		no	yes	
	no	9	1	
	yes	3	10	82.6%
Harding		no	yes	
	no	25	3	
	yes	4	2	79.4%

### Validation of the predictive algorithm by *in vitro* testing

**Fig 5:**
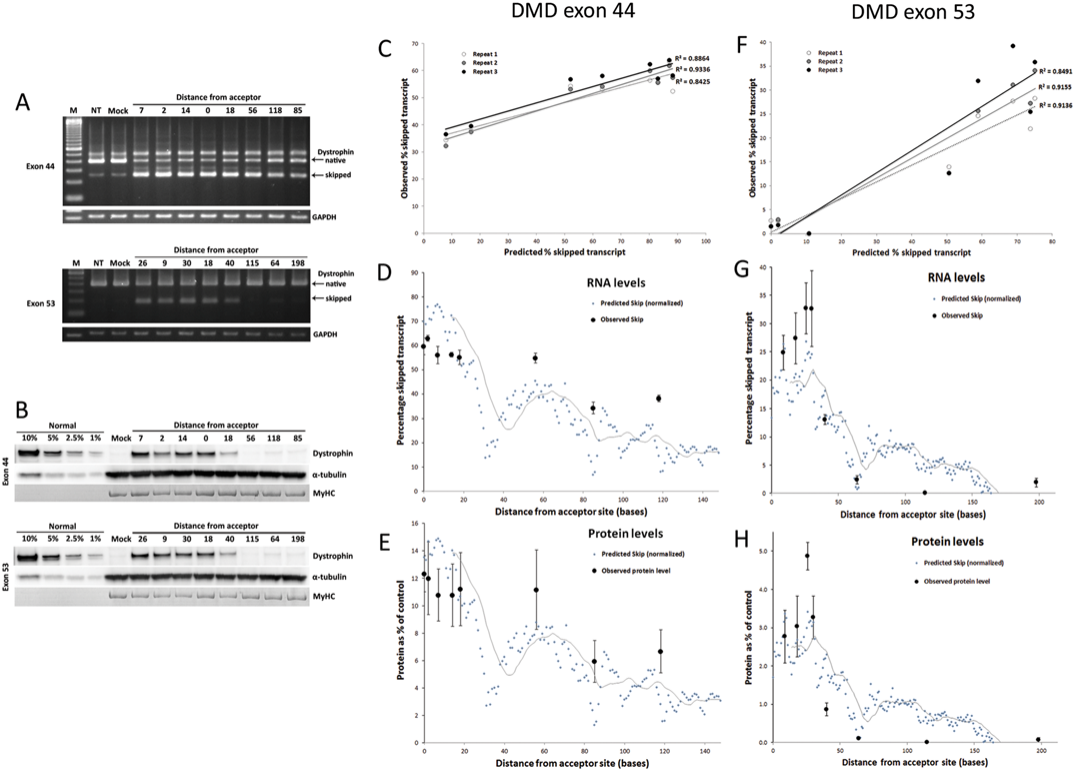
Prospective testing of new PMO oligo sequences targeting exons 44 and 53 of the human dystrophin transcript. (A) Representative gels showing RT-PCR of the native and exon-44-skipped (252 bp) or exon-53-skipped (190 bp) transcripts following exon skipping treatments in cell lines derived from a DMD patient harbouring targetable mutations in exons 44 or 53. M: 100 bp ladder, NT: non-treated, Mock: random 31-mer; test oligos are numbered according to their distance from the acceptor site. As a loading control, GAPDH is shown. (B) Western blots using an anti-dystrophin C-terminal antibody, showing rescued truncated dystrophin protein. A calibration curve of full-length dystrophin from normal control cells was loaded for comparison; Mock: random 31-mer PMO; test oligos are numbered according to their distance from the acceptor site; MyHC: Myosin Heavy Chain loading control. (C-E, F-H) Quantification of RNA and protein levels for exons 44 and 53, respectively. (C, F) Observed skipping efficacy (skipped transcript as a percentage of total non-skipped plus skipped transcript) is plotted against predicted values. R^2^ values are shown for each repeat. (D-E, G-H) Plots of predicted skipping efficacy against distance from exon acceptor site, showing observed skipped transcript levels (D, G) or observed protein levels (E, H). Values for predicted skip are normalized to the average value of the observed skips or of the observed protein levels, to allow for inter-study variation in general skipping efficacy. Distance from acceptor is given for the first (5’-most) base of the target site. A moving average is shown over 15 bases (in this way, the moving average indicates the value for the mid-point of each target site).

We tested new sequences targeting sixteen positions spread across exons 44 and 53 of the human dystrophin transcript, these positions representing a wide range of predicted skipping efficacy according to our algorithm for PMOs (S4 Table). In three experimental repeats, skipped transcript and rescued (truncated) protein levels were measured in treated, untreated, and mock (random 25 or 31-mer)-treated, immortalized cell lines derived from muscle of two DMD patients harbouring appropriate mutations (representative gels are shown in Fig. 5A and 5B; all gels are shown in S5 and S6 Figs.). We observed very strong correlation of observed levels of skipped transcript with predicted skipping efficacy (Fig. 5C and 5F; average R^2^ 0.89 for both exons). Observed protein levels also correlated strongly with prediction (average R^2^ of 0.73 for exon 44 and of 0.78 for exon 53). Due to inter-study variation, our test sequences, despite being PMOs of length 30-mer, cannot be compared directly with the PMO dataset on which the predictive formula is based (for example, we amplified transcripts using a standard single step of PCR rather than nested PCR, we used unmodified bare-morpholinos as opposed to leashed oligos, and the end-porter transfection reagent as opposed to lipotransfection). Due to these differences, when plotting these data against distance from acceptor for each exon we normalized the predicted skip to the average of the observed skips or of the observed protein levels for the test oligos (Fig. 5D-E and 5G-H). For exon 44, a low level of native skipped transcript was observed in untreated controls—this level was subtracted to quantify skipping in the treated samples. For both exons, a clear pattern is apparent matching observations with predictions across the length of the exon. Greatest efficacy was observed for the oligos targeting positions 0 and 2 (exon 44) or 26 and 30 (exon 53) bases away from the splice acceptor site, within regions of high predicted efficacy.

Since our predictive model was constructed using data that included exons 44 and 53, we wished to guard against the possibility that our model was biased towards these exons, and that this would explain the strength of correlation between our experimental data and the predictive algorithm. For this we repeated statistical modelling of the PMO dataset, based on the same four predictive parameters, but excluding either exon 44 or 53 (S7 and S8 Figs.). With exon 44 or 53 omitted, prediction of percentage skip (using a standard least squares model) gave similar R^2^ (0.51 and 0.60, respectively) compared to modelling on the full PMO dataset (0.57; S7 and S8 Figs., A, upper panel), and categorization into > 30% skip or < 30% skip (using an ordinal logistic model) was also similar (correct placement for 96% and 86% of oligos, respectively; S7 and S8 Figs., A, lower panel) to the model for the full PMO dataset (89%). Likewise, very strong correlation of our experimentally observed levels of skipped transcript with predicted skipping efficacy was preserved (S7 and S8 Figs.; average R^2^ of 0.89 and 0.92, respectively).

*In silico* screening tool: The process described above to screen across a *DMD* exon, predicting efficacy for each target site, can be reproduced for any target exon sequence. We provide instructions for how to implement such a screen (see materials and methods), using our Perl code, the RNAstructure software [41], and a spreadsheet program such as Microsoft Excel.

## Discussion

Robust predictive algorithms for the design of splice-switching oligonucleotides would help to optimize this approach in pathologies for which it is already being tested, and help facilitate its application to a large range of diseases that feature RNA mis-splicing. The scope of our study is limited to the DMD pathology and to the PMO and 2’O-Methyl chemistries that are currently being explored in clinical trials, but it may nevertheless inform efforts towards general prediction, Our *in silico* screening tool may help experimenters to choose which target sites to test *in vitro*, and this could reduce the number of oligos that must be tested to achieve satisfactory efficacy.

We focused our experimental validation of the models on the PMO chemistry and on two exons (exons 44 and 53 of *DMD*), measuring both mRNA and protein levels for three experimental repeats of each tested oligo, because we wished to show clearly whether predicted trends within the exon were respected by the experimental data. Our validation establishes this approach for *DMD*, but future studies will be required to determine whether predictions are as accurate for the exons of other genes. This will require large datasets from multiple pathologies and thus is a long-term challenge dependent on the continued output of the wider exon skipping community. Of the models for PMO and 2’O-Methyl data the former provides the better fit, so it will be important in future efforts to refine the 2’O-Methyl model using new data and/or new predictive parameters. For the purposes of data modelling, we would encourage our colleagues in the field to report skipping efficacy as a continuous variable (e.g. the precise percentage of the skipped transcript, as reported in the Popplewell *et al*. PMO dataset and the Dwi Pramono *et al*. 2’O-Methyl dataset) rather than an ordinal variable (such as the skipping levels reported for the majority of 2’O-Methyl studies).

Our most striking observation was that, despite analysing a large number of descriptors of the exon skipping system, it was not useful to include more than 4 parameters into predictive models. For both PMO and 2’O Methyl chemistries, the dG of binding of the oligo to the target region and the distance of the target from the exon acceptor site were strongly predictive. After inclusion of those into the statistical models, addition only of the Malueka splice type of the exon for PMO efficacy prediction [38], and of a simple descriptor of oligo length for 2’O Methyl efficacy prediction, were beneficial to predictive strength. These parameters together were of sufficient predictive power that they could correctly distinguish 89% of PMO and 76% of 2’O Methyl oligos into high and low efficacy groups. The inability of other parameters to much improve K-fold R^2^ suggests that the remaining variance of the exon skipping system is highly stochastic and inherently unpredictable or that we do not understand it sufficiently to define and compute additional predictive parameters.

The Malueka exon type C is characterized by strongly defined but low GC content acceptor sites, and a low density of ESS motifs, and was associated in the PMO dataset with greater efficacy than type A which is characterized by a high presence of SF2/ASF ESE motifs. In contrast, the Malueka exon type parameter was not predictive of efficacy in the 2’O Methyl datasets, and none of our splice motif parameters (including scores for both SF2/ASF ESEs and ESSs), nor our calculation of upstream GC content improved our predictive models for either chemistry. Hence, although we have included it in our model for PMO efficacy, we are hesitant to put it forward as a general predictive parameter, and would wait for future studies on both PMOs and other chemistries before drawing that conclusion. In any case, our model suggests that the Malueka category affects comparisons between exons rather than the relative efficacy of different target sites in the same exon. Cross-terms of the Malueka category with other parameters (such as binding energy or distance from acceptor) did not help for prediction. As such, although we retain the Malueka category function in the algorithm, it could be safely ignored when the aim (as in this work) is to identify the better target sites within a given exon.

The inclusion of an oligo length category into the model for 2’O Methyl oligos even after the modelling of binding energy may suggest the importance of some mechanism that is influenced by length and that is independent of energetics or, more likely, the linear regression algorithm finding yet more leverage on binding energy via the latter’s relationship to length (i.e. the length parameter serving as a subsidiary index of binding energy). In other words, length may be a useful predictive parameter whilst also acting as little more than a crude approximation of binding energy. It should be understood that, in this type of analysis, other parameters may be strongly predictive but are not selected because they do not add to the predictiveness after the inclusion of related but more strongly predictive parameters. For example, in modelling of the PMO dataset: length of the oligo was not useful after the dG of binding had already been modelled, and distance from acceptor to the centre of the target (the ACP value; [30]) was not useful after distance from acceptor to the nearest base of the target had already been modelled.

Both binding energetics and distance from acceptor site have previously been identified to discriminate effective from non-effective oligos [28,29]. All of the descriptors of binding energetics that we considered were predictive, but we favoured the use of dG50 (the energy of oligo binding to the target plus 50 base flanks), a parameter not studied previously, in our final predictive models because this was superior to other parameters for the 2’O-Methyl data and only marginally inferior to the oligo::exon binding energy for the PMO data. It should be noted that the thermodynamics computations are based on RNA to RNA binding, and may be improved if algorithms tailored to these other chemistries were to become available. The usefulness of the distance from acceptor parameter was noted in previous studies and was suggested to be related to the timing of splicing events: the oligo would have more chance to bind the exon prior to exon definition if it targets the portion of the exon that is first transcribed [28,35]. Structural effects arising from the tertiary complex formation of the upstream spliceosome, adjacent to the acceptor site, might also be considered if suitable algorithms were available.

Despite the importance of target site accessibility to siRNA efficacy [46,47], we could not identify a predictive descriptor of this in exon skipping, although some aspects of RNA accessibility are likely represented in the calculation for binding energy to the target with 50 base flanks, which was strongly predictive. It should be borne in mind that the thermodynamic computations of folding assume a single free-floating molecule of RNA, and no attempt is made to account for the cellular environment: potential effects of nearby transcriptional machinery, spliceosomal complex(es) and splicing regulatory factors are not currently possible to compute. Even with the window of computation constrained as it was to a region of 150 bases, local folding could still be influenced by these unknown factors, rendering the computed accessibility less accurate. This could be a particular problem in the context of exon skipping as opposed to RNA interference since mature mRNA may be less constrained by bound proteins. A much deeper understanding of splicing and in particular the quaternary structure dynamics would be required before such factors could be taken into account in computation.

Despite the apparent importance that differential GC content between the intron and exon has to exon definition [39], it does not appear to interact with skipping efficacy, perhaps due to a lack of sequence-specific effects. The neighbourhood inference score for the similarity of a sequence to known splice motifs [37], despite its promise as a general tool to identify splice factor binding sites, was likewise not predictive.

In our modelling to identify the strongest predictors of efficacy, when applied to the Aartsma-Rus dataset alone and to categorize as those authors did in their study into >5% or <5% skipping, we identified the same parameters (binding energy, the number of RESCUE-ESE hexamers, Tra2β and SC35 scores) as those authors did (except that in our models, the inclusion of SC35 scores had no effect on the percentage correct classification; data not shown). However, when we modelled the categorization of these oligos into their reported skipping levels (>25%, 1–25%, undetected) neither the number of RESCUE-ESE hexamers, nor Tra2β scores, nor SC35 scores improved the Bayesian information criterion (BIC) of the model. Thus we did not consider these parameters useful to include into our predictive algorithm. Similarly, none of these parameters were predictive for the Popplewell PMO dataset. Certain other parameters were also not additive to the predictive power of our models despite being capable to distinguish efficacy levels in previous studies: these included hybridisation peaks and SF2/ASF splicing motif scores [29], and co-transcriptional RNA accessibility prediction [35]. As with the Aartsma-Rus dataset, we could confirm that these parameters were predictive in the context of their original studies, but in our modelling analyses they were overshadowed by more powerfully predictive parameters such as binding energetics and the proximity of the target site to the splice acceptor. In fact, we identified no splice motif related parameter that was useful in this sense.

As noted previously, the algorithms used to predict splicing motifs do not take into account the local RNA secondary structure, nor the effect that local oligo binding might have on this structure [29]—perhaps if some aspect of folding could be incorporated into future forms of these algorithms then they may become more strongly predictive. Likewise, improved understanding of structural recognition on the part of specific splicing motifs may also aid prediction. The scores derived from computations of co-transcriptional folding dynamics were previously shown to discriminate certain levels of efficacy in the Aartsma-Rus and Wilton datasets [35], but when applied collectively to those two datasets in our modelling analyses those parameters were entirely overshadowed by dG of binding, and their inclusion did not improve the Bayesian information criterion (BIC) of the model. Notably, it was recently found that local predictions of RNA folding are more accurate than global predictions, the authors giving an optimal window of 150 bases as a reasonable balance between maximizing accurate prediction of base pairing, while minimizing effects of incorrect long-range predictions [36]. This may explain why co-transcriptional RNA accessibility scores, which consider a folding window of 1500 bases, were not additive over local computations of binding energetics.

We note that the datasets on which our models are based are dominated by oligos targeting exonic sequences, and a dataset describing many oligos that target the splice site boundary, Wilton *et al*. [45], is omitted for reasons described. For this reason, we limited predictive analyses to exonic regions, which is in keeping with the observation of Aartsma-Rus *et al*. that exonic regions give greater efficacy [31]. Our predictive algorithms should not be applied to intronic sequences because certain parameters may behave quite differently in the intronic context (such as the distance from acceptor, which changes its sign, becoming negative in the upstream intron, thereby inverting its contribution to the predictive formula).

One can imagine other factors that could influence exon skipping efficacy but that we have not assessed (often because estimates would presently be difficult or impossible to calculate), such as the rates of oligo breakdown, expulsion, and recycling (propensity of an oligonucleotide molecule to be retained during successive rounds of transcription) in target cells, or the capacity of a given sequence to penetrate through the cell and reach the splicing machinery of the nucleus. This last parameter would itself depend on complex factors relating to the chemistry used (diffusion rates within different cell compartments; propensity to bind the chemical groups of other molecules) and perhaps to the specific sequence (‘off-target binding’: the propensity to partially or fully bind native nucleotidic sequences, and the frequency of those sequences). It is also conceivable that the specific mutation of the patient, by changing the folded structure or some other characteristic of the sequence neighbouring the target exon, could affect the efficacy of skipping in a target-dependent manner. Epigenetic factors such as histone modifications are also known to influence splicing (reviewed [48]). For *in vivo* administration, yet more factors may come into play, such as tissue-specific absorption, or rate of elimination by the kidneys and other organ ‘sinks’, though it is perhaps not expected to find strong sequence-specific influences on these parameters, them being decided chiefly by the chemistry used, or by the vector in the case of viral delivery vectors such as AAV [49]. Tissue specific effects are of great importance to therapeutic potential because certain chemistries are more capable than others to target the heart and other affected muscles [50]. It could be hoped that future studies will provide more empirical data on which to base estimates of these factors, for example such as the ongoing work on the cellular uptake mechanisms of peptide-conjugated PMOs [51,52], and the recent finding that PMOs *in vivo* are preferentially taken up by regenerating myofibres [53].

It is widely accepted within the exon skipping field that comparisons of efficacy cannot be made between experiments and can only be made within an experiment. Certainly, for our predictions derived from previous PMO data, it is clear from their comparison with observed skipping values in our prospective PMO study, and their comparison with previous observed skipping values in 2’O-Methyl studies, that study to study variation renders the absolute value of predicted skip to be largely incomparable between studies. However, the relative skipping percentages observed within each study, and within each exon for a given study, appear to be well conserved. It is this relative value that is of importance in the design of new oligos to target a given exon. Exon definition during splicing has been described as a system ‘on the edge of chaos’ [54]: within pre-mRNA, potential splice sites are some 10-fold more numerous than those actually used, and potential regulatory elements abound. This is how single base mutations can switch splicing. The destruction of this definition by the use of antisense oligonucleotides may therefore rely on subtle nuances of the system, in addition to the more powerful influences already identified here and in previous studies: chiefly binding energetics and the distance of the target from the acceptor site. Splicing motifs and RNA accessibility may rank among the more subtle influences, explaining why certain of these parameters are observed to have predictive power in specific studies. Improved algorithms for the computation of these parameters and of binding energetics, especially tailored towards specific chemistries, could help prediction.

Our modelling and validation were restricted to the exons of *DMD*. Even within that scope, due to the inherent variability of the system, and the inability to model all its aspects, it is unlikely that our predictive algorithms would suffice alone to be certain of identifying the optimal target site for a given exon. In addition, the model derivation should be revisited as more data become available, especially for exons of genes other than *DMD*. However, it is hoped that the formulae presented here, when applied as an *in silico* pre-screen across each potential target site, may allow researchers to prioritize favourable regions of the exon for *in vitro* testing, and thereby reduce the number of *de novo* sequences that must be tested in order to identify one or more of high efficacy. Indeed, in applying the pre-screening process to *DMD* exon 53, we identified a potential candidate oligonucleotide (Fig. 5; targeting 26 bases from the acceptor site) for clinical trials. Such screening will be useful not only for previously untargeted exons of *DMD* and other genes, but also for exons that are already targeted in clinical trials, since the predictive algorithm may suggest previously overlooked regions of the target exon. It is our intention in future work to provide a public web interface for access to the *in silico* screening tool.

## Supporting Information

S1 Fig Reported skipping levels for all 79 *DMD* exons targeted by Wilton study. Level 0 = <10%; level 1 = 10–30%; level 2 = >30%. Skipping levels were generally low for exons 55–70 relative to other regions of the gene.
(TIF)

S2 Fig Formula from ordinal logistic model to categorize exon efficacy, based on 2’O Methyl data. (TIF)

S3 Fig Exon screening for the 2’O-Methyl chemistry, showing predicted exon skipping and comparison with published data. Distance from acceptor is given for the first (5’-most) base of the target site. Because these datasets generally reported skipping levels rather than an exact value, the model predicts the probability of a ‘good’ skip (greater than 25%, 27.5%, or 30% skipping, depending on the stratification of skip levels reported in the study) as opposed to predicting the skipping value (such as was possible for the PMO dataset in Fig. 3). The probability of a ‘good’ skip is shown for 20-mer and 25-mer oligonucleotides, with moving averages shown over 10 and 12.5 bases, respectively (in this way, the moving average indicates the value for the mid-point of each target site). These two lengths were chosen to represent the range of lengths used in the 2’O-Methyl datasets (see S4 Fig.). Vertical lines indicate observations from previous studies: green and grey lines represent ‘good’ or ‘bad’ skipping, respectively.
(TIF)

S4 Fig Scatterplot showing the lengths of oligos used in previous 2’O-Methyl studies, separated by author and target exon. Levels of skipping reported as ‘good’ (greater than 25%, 27.5%, or 30% skipping, depending on the stratification of skip levels reported in the study) are indicated by red points, the others in blue.
(TIF)

S5 Fig Uncropped images of the gels and blots relating to prospective testing of new PMO oligo sequences of length 30 bases against *DMD* exon 44 (data presented in Fig. 5). (A) gels showing RT-PCR of the native and exon-44-skipped (252 bp) transcripts following three separate exon skipping treatments in cell lines derived from a DMD patient harbouring an exon 44 targetable mutation. M: 100 bp ladder, NT: non-treated, Mock: random 31-mer PMO; test oligos are numbered according to their distance from the acceptor site. (B) Western blots using an anti-dystrophin C-terminal antibody, showing rescued truncated dystrophin protein. A calibration curve of full-length dystrophin from normal control cells was loaded for comparison; Mock: random 31-mer PMO; test oligos are numbered according to their distance from the acceptor site.
(TIF)

S6 Fig Uncropped images of the gels and blots relating to prospective testing of new oligo sequences against *DMD* exon 53 (data presented in Fig. 5). (A) gels showing RT-PCR of the native and exon-53-skipped (190 bp) transcripts following three separate exon skipping treatments in cell lines derived from a DMD patient harbouring an exon 53 targetable mutation. M: 100 bp ladder, NT: non-treated, Mock: random 31-mer PMO; test oligos are numbered according to their distance from the acceptor site. (B) Western blots using an anti-dystrophin C-terminal antibody, showing rescued truncated dystrophin protein. A calibration curve of full-length dystrophin from normal control cells was loaded for comparison; Mock: random 31-mer PMO; test oligos are numbered according to their distance from the acceptor site.
(TIF)

S7 Fig Re-modelling of PMO dataset omitting exon 44. Models used here were based on the same four parameters as were used for the predictive model against which our experimentally observed values from prospective testing are plotted in Fig. 5. (A) Upper panel: reported skip versus skip predicted in standard least squares model, showing R2 of 0.51; Lower panel: confusion matrix for ordinal logistic model, showing correct categorization for 96% of oligos. (B) Predicted skip from least squares model and observed skip for each experimental repeat, each plotted against distance from acceptor; (C) Observed skipped transcript levels against predicted skip for each experimental repeat.
(TIF)

S8 Fig Re-modelling of PMO dataset omitting exon 53. Models used here were based on the same four parameters as were used for the predictive model against which our experimentally observed values from prospective testing are plotted in Fig. 5. (A) Upper panel: reported skip versus skip predicted in standard least squares model, showing R2 of 0.6; Lower panel: confusion matrix for ordinal logistic model, showing correct categorization for 86% of oligos. (B) Predicted skip from least squares model and observed skip for each experimental repeat, each plotted against distance from acceptor; (C) Observed skipped transcript levels against predicted skip for each experimental repeat.
(TIF)

S1 Materials and Methods Supplemental materials and methods. (DOCX)

S1 Script Perl script for *in silico* screening to predict exon skipping efficacies at all positions of a target exon. (PL)

S1 Sequences Sequences of human *DMD* exons 44 to 55, with 200 base flanking regions, in fasta format. (FASTA)

S1 Table Compiled data of tested PMO sequences with published and newly calculated parameters. (XLSX)

S2 Table Compiled data of tested 2’O-Methyl sequences with published and newly calculated parameters. (XLSX)

S3 Table Parameters tested in statistical modelling. ^†^Predicted skip is shown for oligos categorised as ‘good’ or not, based on the level stratifications reported for each study (Aartsma-Rus, >25%; Dwi Pramono, >27.5%; Harding, >30%), green background = correctly predicted; red = incorrectly predicted.
(TIF)

S4 Table Description of oligos used in prospective testing. (DOCX)
