# Supplementary figures and images for: In Silico Screening Based on Predictive Algorithms as a Design Tool for Exon Skipping Oligonucleotides in Duchenne Muscular Dystrophy
Source: PLoS One. 2015 Mar 27;10(3):e0120058. doi: 10.1371/journal.pone.0120058 (PMC4376395; doi:10.1371/journal.pone.0120058)

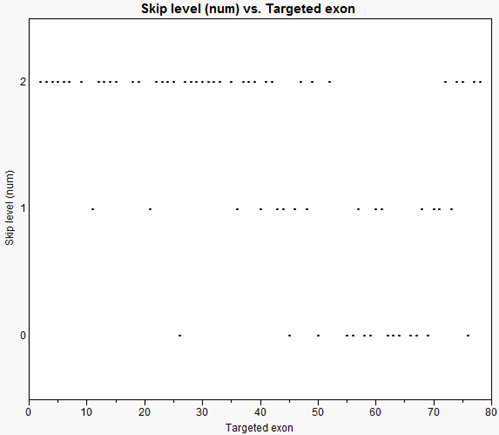

Supplement: S1 Fig — Level 0 = <10%; level 1 = 10–30%; level 2 = >30%. Skipping levels were generally low for exons 55–70 relative to other regions of the gene. (TIF) [file pone.0120058.s001.tif]

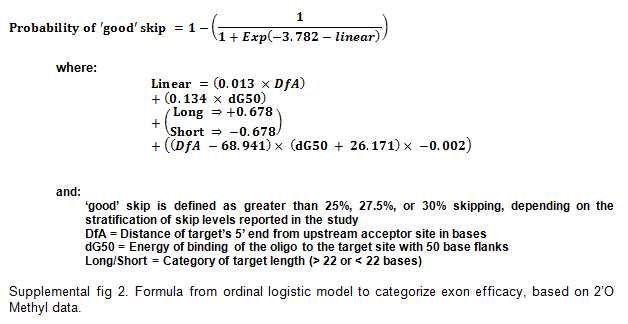

Supplement: S2 Fig — (TIF) [file pone.0120058.s002.tif]

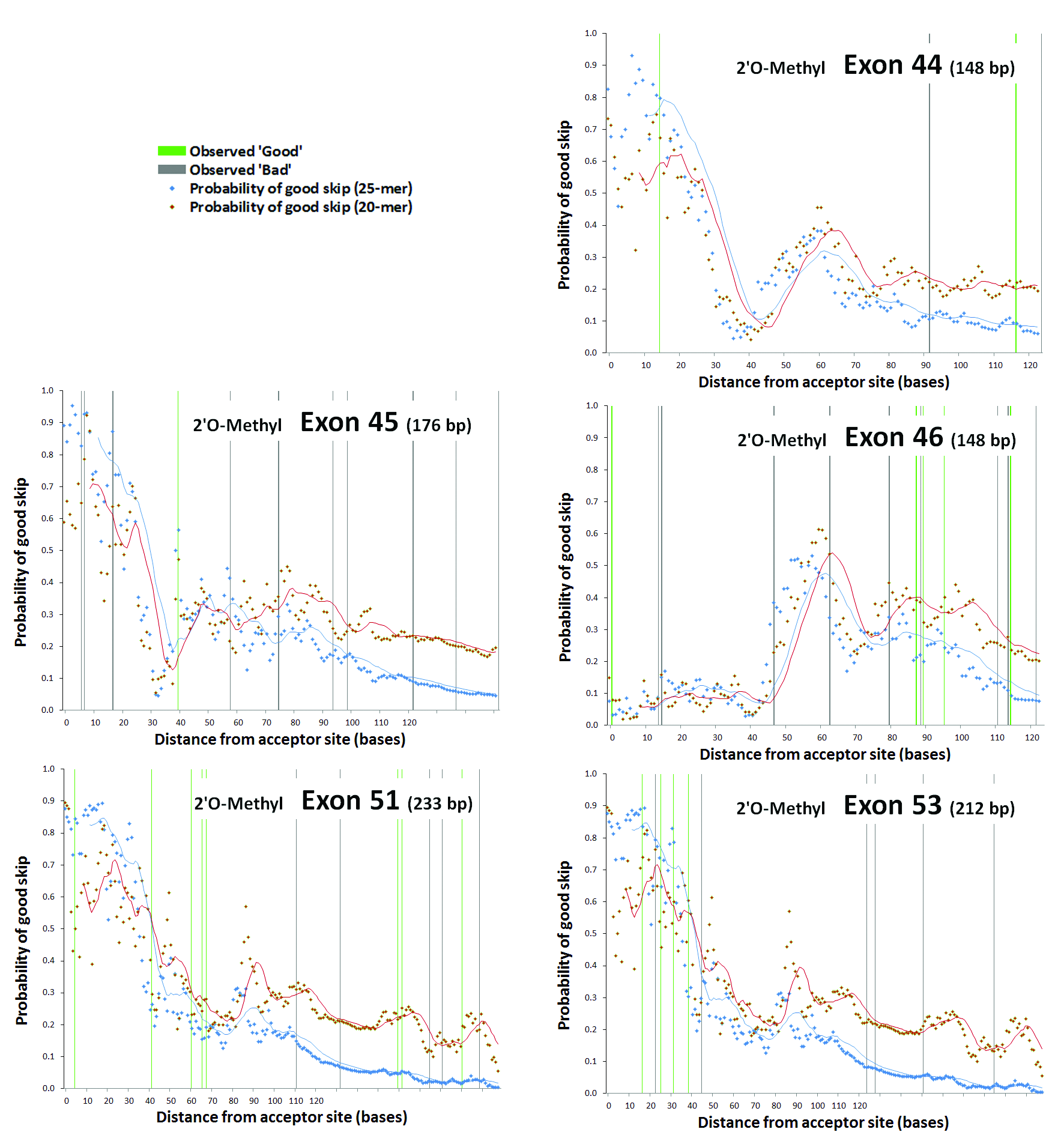

Supplement: S3 Fig — Distance from acceptor is given for the first (5’-most) base of the target site. Because these datasets generally reported skipping levels rather than an exact value, the model predicts the probability of a ‘good’ skip (greater than 25%, 27.5%, or 30% skipping, depending on the stratification of skip levels reported in the study) as opposed to predicting the skipping value (such as was possible for the PMO dataset in Fig. 3). The probability of a ‘good’ skip is shown for 20-mer and 25-mer oligonucleotides, with moving averages shown over 10 and 12.5 bases, respectively (in this way, the moving average indicates the value for the mid-point of each target site). These two lengths were chosen to represent the range of lengths used in the 2’O-Methyl datasets (see S4 Fig.). Vertical lines indicate observations from previous studies: green and grey lines represent ‘good’ or ‘bad’ skipping, respectively. (TIF) [file pone.0120058.s003.tif]

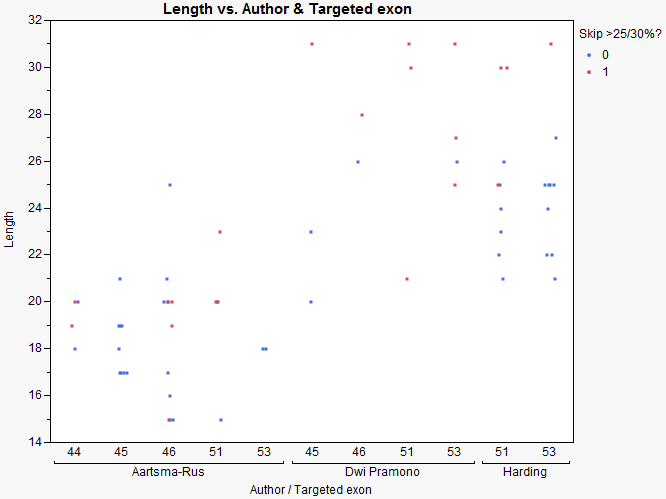

Supplement: S4 Fig — Levels of skipping reported as ‘good’ (greater than 25%, 27.5%, or 30% skipping, depending on the stratification of skip levels reported in the study) are indicated by red points, the others in blue. (TIF) [file pone.0120058.s004.tif]

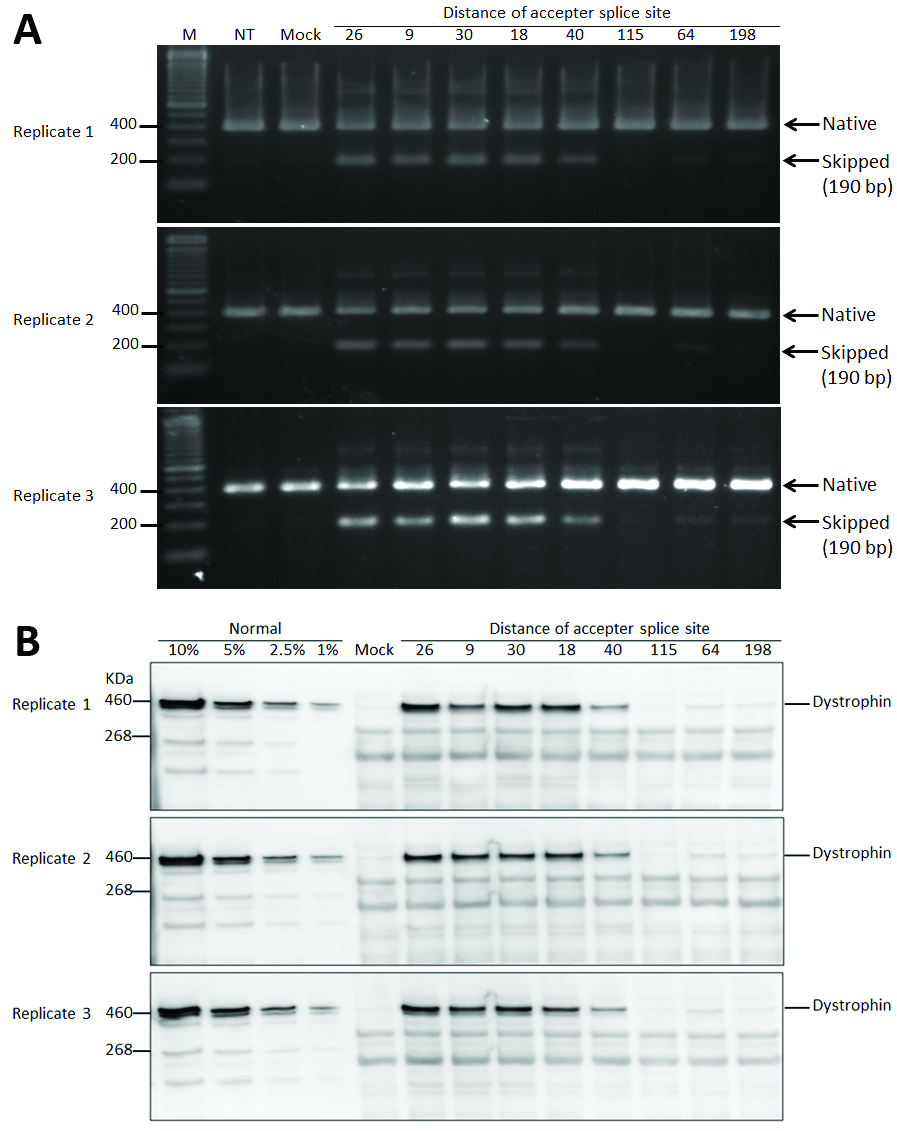

Supplement: S5 Fig — (A) gels showing RT-PCR of the native and exon-44-skipped (252 bp) transcripts following three separate exon skipping treatments in cell lines derived from a DMD patient harbouring an exon 44 targetable mutation. M: 100 bp ladder, NT: non-treated, Mock: random 31-mer PMO; test oligos are numbered according to their distance from the acceptor site. (B) Western blots using an anti-dystrophin C-terminal antibody, showing rescued truncated dystrophin protein. A calibration curve of full-length dystrophin from normal control cells was loaded for comparison; Mock: random 31-mer PMO; test oligos are numbered according to their distance from the acceptor site. (TIF) [file pone.0120058.s005.tif]

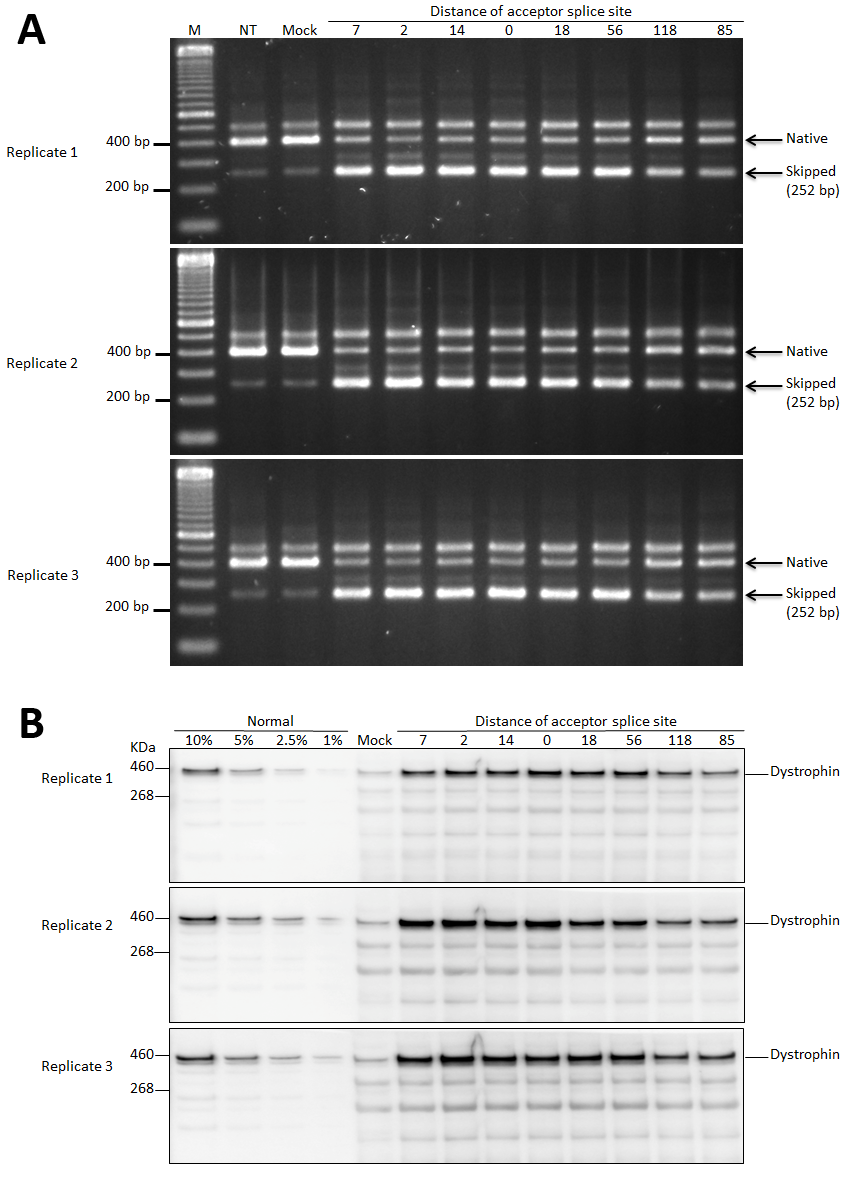

Supplement: S6 Fig — (A) gels showing RT-PCR of the native and exon-53-skipped (190 bp) transcripts following three separate exon skipping treatments in cell lines derived from a DMD patient harbouring an exon 53 targetable mutation. M: 100 bp ladder, NT: non-treated, Mock: random 31-mer PMO; test oligos are numbered according to their distance from the acceptor site. (B) Western blots using an anti-dystrophin C-terminal antibody, showing rescued truncated dystrophin protein. A calibration curve of full-length dystrophin from normal control cells was loaded for comparison; Mock: random 31-mer PMO; test oligos are numbered according to their distance from the acceptor site. (TIF) [file pone.0120058.s006.tif]

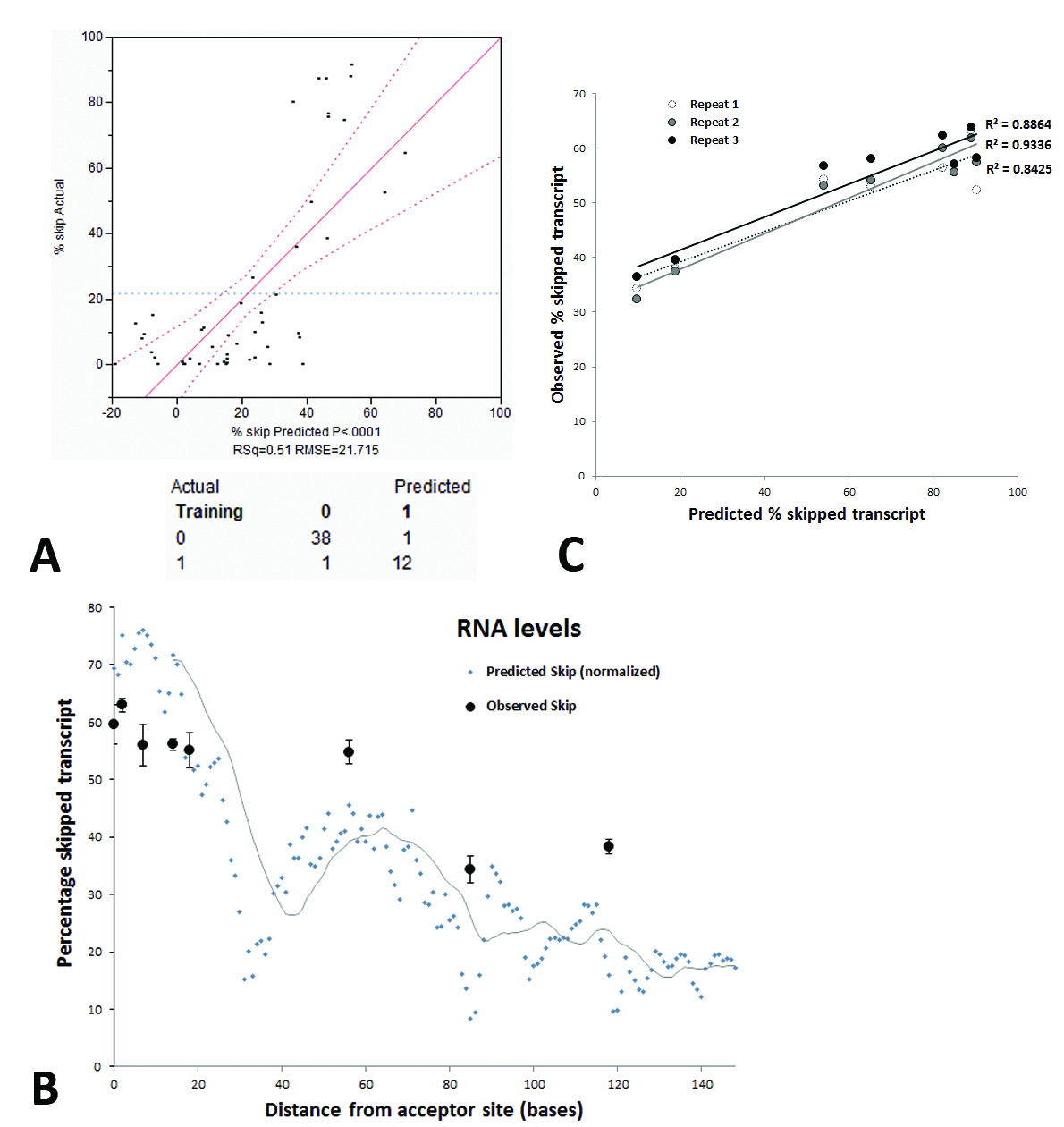

Supplement: S7 Fig — Models used here were based on the same four parameters as were used for the predictive model against which our experimentally observed values from prospective testing are plotted in Fig. 5. (A) Upper panel: reported skip versus skip predicted in standard least squares model, showing R2 of 0.51; Lower panel: confusion matrix for ordinal logistic model, showing correct categorization for 96% of oligos. (B) Predicted skip from least squares model and observed skip for each experimental repeat, each plotted against distance from acceptor; (C) Observed skipped transcript levels against predicted skip for each experimental repeat. (TIF) [file pone.0120058.s007.tif]

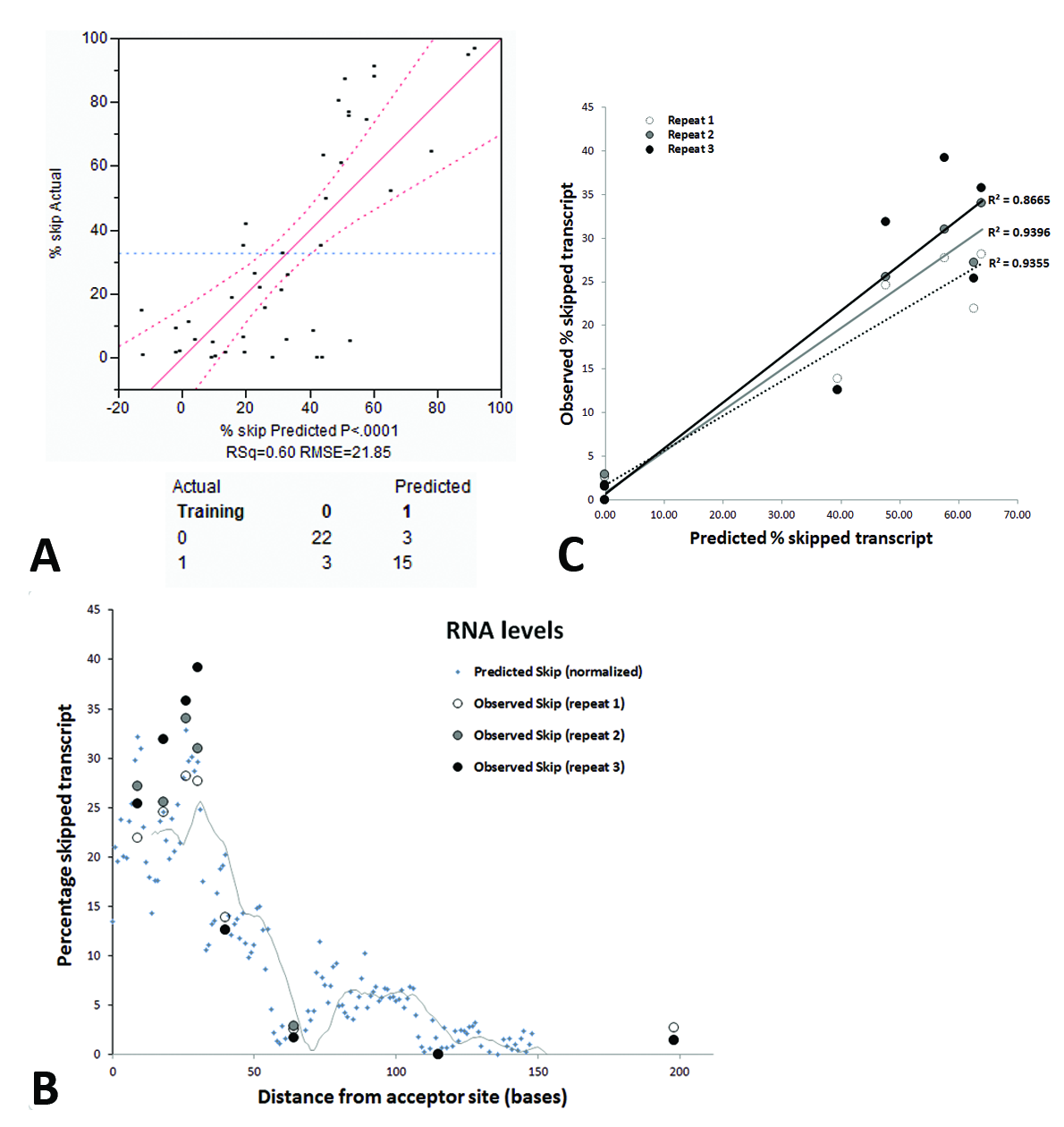

Supplement: S8 Fig — Models used here were based on the same four parameters as were used for the predictive model against which our experimentally observed values from prospective testing are plotted in Fig. 5. (A) Upper panel: reported skip versus skip predicted in standard least squares model, showing R2 of 0.6; Lower panel: confusion matrix for ordinal logistic model, showing correct categorization for 86% of oligos. (B) Predicted skip from least squares model and observed skip for each experimental repeat, each plotted against distance from acceptor; (C) Observed skipped transcript levels against predicted skip for each experimental repeat. (TIF) [file pone.0120058.s008.tif]

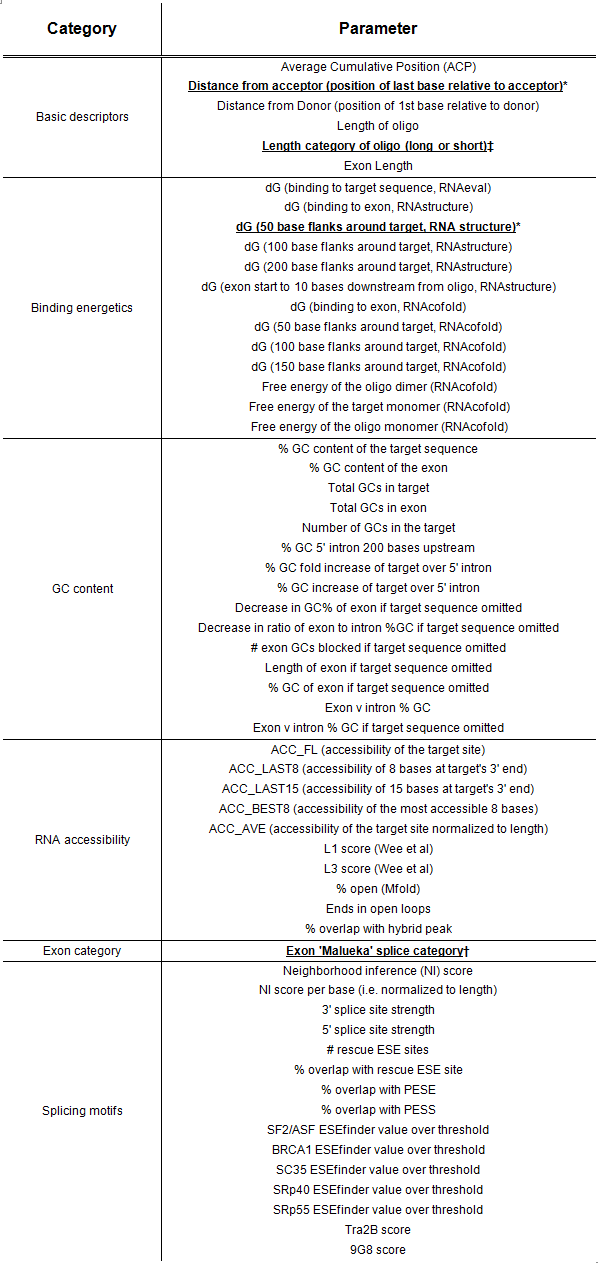

Supplement: S3 Table — †Predicted skip is shown for oligos categorised as ‘good’ or not, based on the level stratifications reported for each study (Aartsma-Rus, >25%; Dwi Pramono, >27.5%; Harding, >30%), green background = correctly predicted; red = incorrectly predicted. (TIF) [file pone.0120058.s014.tif]
