# Supplementary material for: In Silico Screening Based on Predictive Algorithms as a Design Tool for Exon Skipping Oligonucleotides in Duchenne Muscular Dystrophy
Source: PLoS One. 2015 Mar 27;10(3):e0120058. doi: 10.1371/journal.pone.0120058 (PMC4376395; doi:10.1371/journal.pone.0120058)
Supplement: S1 Materials and Methods — (DOCX) [file pone.0120058.s009.docx]

**SUPPLEMENTAL MATERIAL AND METHODS**

*Binding energetics:* The ‘bifold’ component of RNAstructure was used (with run parameters -i -L 30 -M 20 -P 10 -T 310.15) to compute ensemble energy for the oligo with the target region, then ‘Fold’ was used for the monomers with default settings, and dG was calculated: ensemble energy – (target energy + oligo energy). RNAcofold was also used to obtain values for the free energy of the target monomer, the oligo monomer, and the oligo dimer. Custom Perl programs (incorporating certain BioPerl functions [42]) were written to automate calculations for the published data.

*RNA accessibility:* RNAplfold algorithm was used with run parameters -noLP -W 150 -L 100 -u 31. The noLP parameter causes structures with lonely pairs (helices of length 1) to be ignored. W and L parameters describe the optimal window size determined by [36]: L sets the maximum distance in bases between paired bases, and W sets the window size over which pairing probabilities are averaged. The u parameter gives output for all sequences up to 31 bases upstream from each target position. RNAplfold was run against all target exons and their flanks.

*Predictive modelling:* To identify strongly predictive parameters: for continuous Y variables, such as the percentage skip reported by [29], we used k-fold cross-validation (with K-fold R^2^ as a stopping rule) with fold number equal to the number of observations in the dataset (this is identical to leave one out cross-validation); for ordinal Y variables, such as skip levels, we used stepwise regression with Bayesian information criterion (BIC) as the stopping rule. These parameters were then used to construct standard least squares or ordinal logistic models from which predictive formulae and confusion matrices were taken. For direct comparisons of predictive power (such as dG50 compared with oligo::target and oligo::exon), values given are the probability of obtaining as large an F Ratio, given that all parameters except the intercept are zero.

*Prospective testing:* RT-PCR was carried out using the SuperScript III one- step RT-PCR system with Platinum Taq DNA polymerase kit (Invitrogen). RNA amounts of 200 ng and 100 ng were used for *DMD* and *GAPDH*, respectively. To detect exon-44-skipped transcript with a 252 bp region and exon-53-skipped transcript with a 190 bp region, the following primer sets were used: forward Ex41_56-75_hDMD_F: sequence AAGCTGAGGGCTTGTCTGAG and Ex45_105-124_hDMD_R: sequence GGCTTCCCAATTTTTCCTGT for exon 44 skipping or forward Ex51_188-207_hDMD_F: sequence GGTGGGTGACCTTGAGGATA and reverse Ex54_125-144_hDMD_R: sequence GCTTCTCCAAGAGGCATTGA for exon-53 skipping. RT-PCR conditions were as follows: 50 ºC 5 m, 94 ºC 2 m, cycle [94 ºC 15s, 60 ºC 30s, 68 ºC 35s], 68 ºC 5 m, with cycle steps numbering 35 or 18 for *DMD* or *GAPDH*, respectively. Electrophoresis was performed with 2% agarose gel and for 5 min at 135 V followed by 20 min at 100 V/gel, and visualized by SYBR safe DNA stain for 30 min (Invitrogen). Five μL of samples were loaded in each well of 5 mm length. PCR products with the expected size for the transcript with exon 44 or 53 deleted were confirmed by direct sequencing. For western blots, protein amount loaded was 12 ug/well. Samples were run in SDS-PAGE for 75 min at 150 V. The semi-dry transfer method was used (70 min at 20 V). Blocking was performed with 2% ECL advance blocking reagent (GE Healthcare), overnight at 4 ºC. The transferred membrane was incubated with primary anti-dystrophin antibodies against the rod domain (NCL-DYS1, 1:400, Leica Microsystems) or the C-terminal domain (ab15277, 1:2500, Abcam) for 1 hour. DYS1 and ab15277 were detected with secondary horseradish peroxidase (HRP)-conjugated anti-mouse Ig2a (1:10000) and HRP-conjugated anti-rabbit IgG H+L (1:10000), respectively. The primary antibodies were visualized with electrochemiluminescence (ECL detection kit, GE Healthcare). Myosin heavy chain was stained with Coomassie Brilliant Blue reagent as a marker of differentiation.
