# Supplementary material for: In Silico Screening Based on Predictive Algorithms as a Design Tool for Exon Skipping Oligonucleotides in Duchenne Muscular Dystrophy
Source: PLoS One. 2015 Mar 27;10(3):e0120058. doi: 10.1371/journal.pone.0120058 (PMC4376395; doi:10.1371/journal.pone.0120058)
Supplement: S4 Table — (DOCX) [file pone.0120058.s015.docx]

| Name | Oligo sequence | Distance from acceptor | Predicted Skip % | Ranking within screen of exon 53 |
| --- | --- | --- | --- | --- |
| hEx53_Ac9-30mer | GTTCTTGTACTTCATCCCACTGATTCTGAA | 9 | 73.9 | 2 |
| hEx53_Ac18-30mer | TCTGAAGGTGTTCTTGTACTTCATCCCACT | 18 | 59.0 | 13 |
| hEx53_Ac26-30mer | CCTCCGGTTCTGAAGGTGTTCTTGTACTTC | 26 | 75.2 | 1 |
| hEx53_Ac30-30mer | GTTGCCTCCGGTTCTGAAGGTGTTCTTGTA | 30 | 69.0 | 7 |
| hEx53_Ac40-30mer | TCATTCAACTGTTGCCTCCGGTTCTGAAGG | 40 | 50.7 | 23 |
| hEx53_Ac64-30mer | CATTGTGTTGAATCCTTTAACATTTCATTC | 64 | 2.1 | 160 |
| hEx53_Ac115-30mer | TTGGCTCTGGCCTGTCCTAAGACCTGCTCA | 115 | 10.9 | 137 |
| hEx53_Ac198-30mer | TATCTTTGATACTAACCTTGGTTTCTGTGA | 198 | -14.4 | 198 |
| hEx44_Ac7-30mer | AAAACGCCGCCATTTCTCAACAGATCTGTC | 7 | 88.19 | 1 |
| hEx44_Ac2-30mer | GCCGCCATTTCTCAACAGATCTGTCAAATC | 2 | 87.06 | 3 |
| hEx44_Ac14-30mer | CATAATGAAAACGCCGCCATTTCTCAACAG | 14 | 83.01 | 7 |
| hEx44_Ac0-30mer | CGCCATTTCTCAACAGATCTGTCAAATCGC | 0 | 80.13 | 12 |
| hEx44_Ac18-30mer | ATATCATAATGAAAACGCCGCCATTTCTCA | 18 | 63.26 | 18 |
| hEx44_Ac56-30mer | ACTGTTCAGCTTCTGTTAGCCACTGATTAA | 56 | 51.96 | 28 |
| hEx44_Ac118-30mer | CTTAAGATACCATTTGTATTTAGCATGTTC | 118 | 16.78 | 135 |
| hEx44_Ac85-30mer | ATTCTCAGGAATTTGTGTCTTTCTGAGAAA | 85 | 7.78 | 199 |

Table S4. Description of oligos used in prospective testing.
